# **Macrophomina phaseolina**: General Characteristics of Pathogenicity and Methods of Control

**DOI:** 10.3389/fpls.2021.634397

**Published:** 2021-04-22

**Authors:** Nathalie Marquez, María L. Giachero, Stéphane Declerck, Daniel A. Ducasse

**Affiliations:** ^1^Instituto de Patología Vegetal, Centro de Investigaciones Agropecuarias, Instituto Nacional de Tecnología Agropecuaria, Córdoba, Argentina; ^2^Consejo Nacional de Investigaciones Científicas y Técnicas (CONICET), Unidad de Fitopatología y Modelización Agrícola (UFYMA), Córdoba, Argentina; ^3^Earth and Life Institute, Mycology, Université catholique de Louvain, Louvain-la-Neuve, Belgium

**Keywords:** *Macrophomina phaseolina*, soil-borne fungus, methods of control, pathogecity, plant pathogen interaction

## Abstract

*Macrophomina phaseolina* is a generalist soil-borne fungus present all over the world. It cause diseases such as stem and root rot, charcoal rot and seedling blight. Under high temperatures and low soil moisture, this fungus can cause substantial yield losses in crops such as soybean, sorghum and groundnut. The wide host range and high persistence of *M. phaseolina* in soil as microsclerotia make disease control challenging. Therefore, understanding the basis of the pathogenicity mechanisms as well as its interactions with host plants is crucial for controlling the pathogen. In this work, we aim to describe the general characteristics and pathogenicity mechanisms of *M. phaseolina*, as well as the hosts defense response. We also review the current methods and most promising forecoming ones to reach a responsible control of the pathogen, with minimal impacts to the environment and natural resources.

## Introduction

*Macrophomina phaseolina* is a generalist soil-borne fungus present all over the world, affecting at least 500 plant species in more than 100 families. It cause diseases such as stem and root rot, charcoal rot and seedling blight (Dhingra and Sinclair, 1978; Ghosh et al., 2018). Under high temperatures (30–35 °C) and low soil moisture (below 60%), this fungus can cause substantial yield losses in crops such as soybean and sorghum, impacting incomes of farmers (Kaur et al., 2012). In the worst case scenario, 100% yield losses have been recorded in groundnut cultivars when disease appeared at pre-emergence stage (Sharma and Bhowmik, 1986).

Despite the many research efforts to control the diseases, the management strategies of *M. phaseolina* remains a challenge. Indeed, diseases caused by this soil pathogen are the result of interactions between the host plant, the pathogen, and the biotic and abiotic components of the environment. Therefore, in this work we aim to (1) describe the general characteristics of *M. phaseolina*, (2) report the most up-to-date knowledge on the pathogenicity mechanisms as well as interactions between the fungal pathogen and its host plants and/or other microorganisms, (3) review the current strategies and most promising forecoming ones to control the pathogen.

## *Macrophomina Phaseolina* General Characteristics

*Macrophomina phaseolina* (Tassi) Goid is a member of the family *Botryosphaeriaceae*. Currently, no subspecies or physiological races, based on morphological or genomic characterizations, have been identified for this fungus (Dhingra and Sinclair, 1978; Crous et al., 2006). However, two new *Macrophomina* species, *M. pseudophaseolina* and *M. euphorbiicola*, have been isolated recently. *M. pseudophaseolina* was isolated from *Abelmoschus esculentus*, *Arachis hypogaea*, *Hibiscus sabdarifa* and *Vigna unguiculata* in Senegal (Sarr et al., 2014) and subsequently in *A. hypogaea*, *Gossypium hirsutum* and *Ricinus communis* and associated with seed decay of *Jatropha curcas* in Brazil (Machado et al., 2019). This fungus appeared to be less distributed than *M. phaseolina* but only slightly differed in pathogenicity (Mbaye et al., 2015). *M. euphorbiicola* was reported as a new phylogenetic species of *Macrophomina* and was found associated with charcoal rot on castor bean (*Ricinus communis*) and bellyache bush (*Jatropha gossypifolia*) in Brazil (Machado et al., 2019).

### Morphological Characteristics

*M. phaseolina* is characterized by hyaline hyphae with thin walls to light brown or dark brown hyphae with septa. Branches from the main hyphae are generally formed at right angle on parent hyphae with constriction at the point of origin. Microsclerotia, a compact mass of hardened fungal mycelium, are spherical, oval or oblong, light brown in the young stage becoming darker (brown to black) with ageing. Pycnidia, which are rarely observed under natural conditions, are larger than microsclerotia, dark brown to black, rough, globose, or irregular, beaked and ostiolated (Lakhran et al., 2018). The fungus can show a wide heterogeneity in mycelium colour, microsclerotia distribution, pycnidia formation and chlorate phenotypes between isolates on synthetic media. Nevertheless, the amplification of the internal transcribed spacers (ITS) has indicated that isolates belonged to one single species (Almomani et al., 2013). It has been suggested that the morphological heterogeneity could be attributed to the responses of the fungi to environmental factors or variation in their hosts species (Tok, 2019; Pandey et al., 2020).

Likewise, a high correlation between virulence and phenotype (i.e., morphological variations) has been reported by Tok (2019).

### Disease Cycle

**FIGURE 1:**
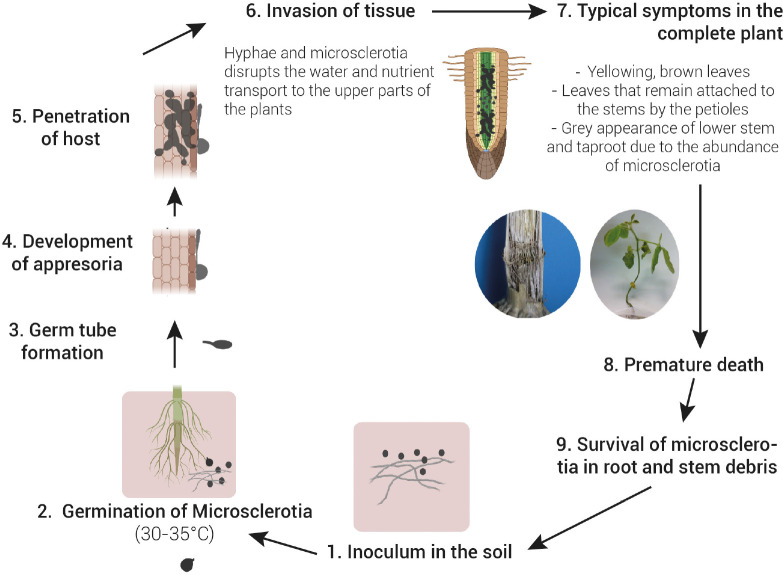
Disease cycle of charcoal rot caused by *Macrophomina phaseolina*. Microsclerotia present in soil is the primary source of inoculum. Microsclerotia germinate (30–35°C) and form a germ tube followed by the development of an appresoria to penetrates through the host epidermis. Once in the roots, the fungus affects the vascular system, disrupting the water and nutrient transport to the upper parts of the plants. This causes wilting of the plant and a typical grey appearance of stem tissues due to the abundance of microsclerotia. Under severe disease and favourable environmental conditions, a premature death of the host plant often occur. Microsclerotia in root and stem debris return to the soil and can either begin a new disease cycle or survive in soil up to 15 years.

**FIGURE 2:**
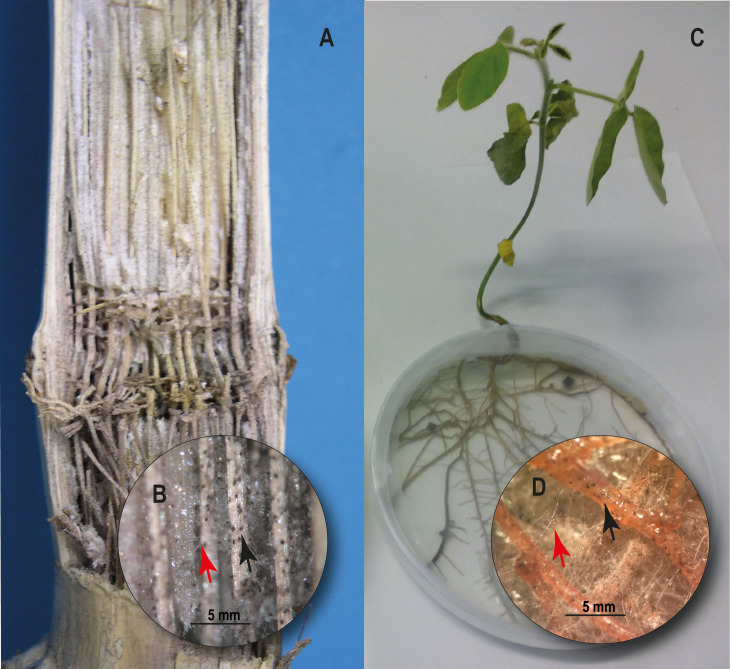
Charcoal rot on corn (*Zea mays* L.) and soybean (*Glycine max*). **(A)** Inside of corn stem showing black discoloration and shredding of vascular bundles. **(B)** *M. phaseolina* hyphae (red arrow) and microsclerotia developed on vascular aces of corn stem (black arrow). **(C)** Soybean plant 10 days post inoculation with *M. phaseolina* under *in vitro* culture conditions. **(D)** *M. phaseolina* hyphae (red arrow) and microsclerotia developed on soybean roots (black arrow).

Microsclerotia is the primary infective source of *M. phaseolina*. This structure of resistance is able to survive up to 15 years in soil (Gupta et al., 2012). It can infect the roots of the host plant at the seedling stage via multiple germinating hyphae. Once in the roots, the fungus affects the vascular system, disrupting the water and nutrient transport to the upper parts of the plants (Figure 1). Typical symptoms are yellowing and senescence of leaves that remain attached to the stems by the petioles, sloughing of cortical tissues from the lower stem and taproot, and the grey appearance of these tissues due to the abundance of microsclerotia that can result in a premature death of the host plant (Short et al., 1978; Wyllie, 1988; Sinclair and Backman, 1989; Smith and Carvil, 1997; Figure 2).

### Genetic Diversity

Genetic diversity among *M. phaseolina* isolates has been widely studied using mostly molecular markers followed by cluster analysis. Genetic methods such as random amplified polymorphic DNA (RAPD), restriction fragment length polymorphism (RFLP), amplified fragment length polymorphism (AFLP) and rDNA sequencing have been successfully used for comparative genomics in *M. phaseolina* population from different countries (Mayék-Pérez et al., 2001; Almeida et al., 2003; Jana et al., 2005; Babu et al., 2010; Khan et al., 2017). Eventhough sexual reproduction in *M. phaseolina* is absent, results showed a high degree of genetic diversity among isolates of this pathogen. It is possible that parasexualism with fusion of cells from different hyphae could occur, and may form heterokaryons that contribute to the variability observed (Almeida et al., 2003).

In some studies (Jana et al., 2005; Babu et al., 2010; Mahdizadeh et al., 2012), genetic diversity has been associated with host plant origin and/or geographical locations, while in other studies (Mahdizadeh et al., 2011; Reznikov et al., 2018, 2019), clustering of data could not clearly differentiate isolates based on their pathogenicity, morphological characteristic, host or geographical origins. In numerous studies the distribution of *M. phaseolina* genotype has been found to be independent of sampling location and host (Khan et al., 2017; Tančić Živanov et al., 2019). Moreover, genetic variability among Brazilian isolates of *M. phaseolina* showed that one single root can harbor more than one haplotype (Almeida et al., 2003). *M. phaseolina* has a very heterogeneous nature. Variation in pathogenicity appeared to be associated with their ability to produce hydrolytic enzymes and to genetic diversity (Ramos et al., 2016; Khan et al., 2017). Thus, attempts to study genotype–genotype specific interactions between plant cultivars and *M. phaseolina* isolates as proposed by Reznikov et al. (2019) will help in the development of resistant cultivars.

### Molecular Diagnostics

Accurate diagnosis and early detection of pathogens is an essential step in plant disease management. Species-specific oligonucleotide primers and oligonucleotide probes can be used to rapidly detect and identify *M. phaseolina* by polymerase chain reaction (PCR) and hybridization (Babu et al., 2007). More recently, specific primers have been developed for the identification of *M. phaseolina*, *M. pseudophaseolina*, and *M. euphorbiicola* (Santos et al., 2020). This may contribute to broader studies conducted to evaluate the diversity and distribution of species of this genus.

Furthermore, a real-time qPCR assay has been developed to detect and quantify *M. phaseolina* abundance in rhizosphere soil and plant tissues. Sets of specific primers have been designed for SYBR green and TaqMan assay (Babu et al., 2011; Burkhardt et al., 2018). These are useful tools for the evaluation of a plant pathogen population in the soil, and it seems possible to estimate the vegetative population of *M. phaseolina* following direct extraction of soil DNA without culturing (Babu et al., 2011).

### Genomic, Proteomic and Metabolic Analysis

In the recent decade, Islam et al. (2012) edited the first whole genome of *M. phaseolina* which was characterized by a large number of enzymes involved in the degradation of cell wall polysaccharides and lignocellulose. This study opened the field to investigate the infection process at the cytological and molecular level via a diverse arsenal of enzymatic and toxin tools infecting a huge diversity of plants. To date and as far as we know, published genomes of *M. phaseolina* include strains isolated from jute, strawberry, alfalfa, and sorghum (Islam et al., 2012; Burkhardt et al., 2019; Quazi et al., 2019; Purushotham et al., 2020).

Recently, proteome data of *M. phaseolina* was provided by Zaman et al. (2020). A total of 2204 proteins were identified, of which 137 were found to be differentially regulated in presence of the biocontrol microorganism *Bacillus contaminans* NZ. Interestingly, most of these proteins with altered expression were related to defense, virulence, cell proliferation, and cell wall composition, together with the proteins of redox and metabolic pathways (Zaman et al., 2020). Interestingly, the metabolites profile of *M. phaseolina* has been compared in the presence and absence of *Eucalyptus globulus* stem tissue (Salvatore et al., 2020). The presence of host tissue during *M. phaseolina* growth induced the production of azelaic acid, suggesting that this secondary metabolite may play a role in disease establishment.

## Pathogenesis of *M. Phaseolina*

**FIGURE 3:**
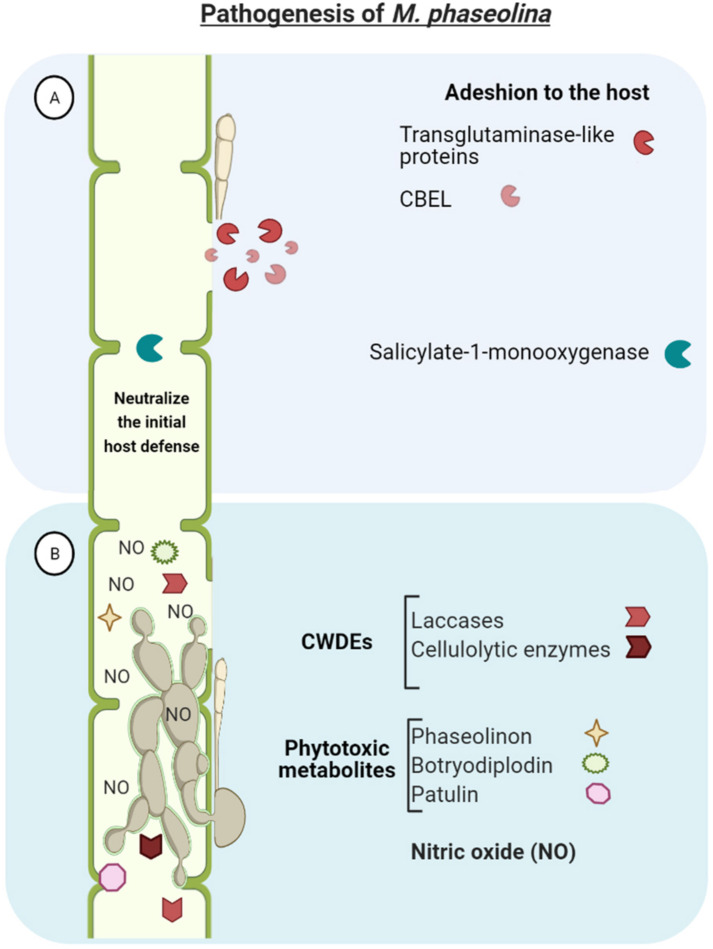
*Macrophomina phaseolina* genome encodes a large repertoire of pathogenicity-associated genes which enables to **(A)** adhere to the host tissue (e.g., CBEL-cellulose binding elicitor lectin and transglutaminase-like proteins), neutralize the initial host defense (i.e., salicylate-1-monooxygenase), and penetrate and invade plant tissues. **(B)** Once in the host, the pathogen releases an array of different toxins and cell wall degrading enzymes (CDWEs) and finally disrupt the vascular system and overthrow host defense, resulting in host cell death and disease establishment.

*M. phaseolina* genome encodes a large repertoire of pathogenicity-associated genes which may be involved in the pathogenesis of the fungus (Figure 3). Actually, 12% of the genes encoded by the genome have similarities with genes involved in pathogen-host interactions. This wide array of genes enables *M. phaseolina* to adhere to the host tissue (i.e., cellulose binding elicitor lectin and transglutaminase-like proteins), neutralize the initial host defense (i.e., salicylate-1-monooxygenase), penetrate and invade plant epidermis. Once in the host, the pathogen releases an array of different toxins and cell wall degrading enzymes (CWDEs) and finally disrupt the host defense, resulting in host cell death and disease establishment (Islam et al., 2012).

A major characteristic of *M. phaseolina* is its large amount of hydrolytic enzymes for degrading cell wall polysaccharides and lignocelluloses to penetrate into the host tissue. This includes: endoglucanases, exocellobiohydrolases, and β-glucosidases for the hydrolysis of cellulose; and laccases, lignin peroxidases, galactose oxidases, and chloroperoxidases, haloperoxidases, and heme peroxidases for lignin degradation. Interestingly, *M. phaseolina* possesses the highest number of laccases and cellulolytic enzymes in comparison with genomes of other sequenced fungal species (e.g., *Postia placenta*, *Phanerochaete chrysosporium, Cryptococcus neoformans, Ustilago maydis, Saccharomyces cerevisia, Aspergillus nidulans*, *Neurospora crassa*) (Islam et al., 2012; Bandara et al., 2018). The production and activity of these plant cell wall-degrading enzymes has been confirmed under *in vitro* culture conditions (Ramos et al., 2016).

Furthermore, phytotoxic metabolites produced by *M. phaseolina* have been described, including phaseolinon, botryodiplodin and patulin, which are believed to play a role in the initial stages of infection, causing wilting of seedlings and formation of necrotic lesions on leaves and roots (Bhattacharya et al., 1987; Ramezani et al., 2007; Abbas et al., 2020; Salvatore et al., 2020). This increases the virulence of *M. phaseolina* and may contribute to explain the highly efficient mechanism to infect different hosts and tissues. The great adaptability of the fungus to a wide range of environmental conditions also contibutes to its ubiquitous distribution and infectivity of plants (Islam et al., 2012; Salvatore et al., 2020). This adaptation consist in the expression of detoxification genes (such as cytochrome P450, Cof, superoxide dismutase) to counter the plant defense response (Ghosh et al., 2018).

Interestingly, *M. phaseolina* genome analysis revealed nitric oxide synthase-like sequence with conserved amino acid sequences. Nitric oxide (NO) was detected *in vitro* inside the mycelium and in the surrounding medium, and in high concentration in infected jute tissues as well. This suggest that NO may have important physiological significance in necrotrophic host pathogen interaction (Sarkar et al., 2014).

Although *M. phaseolina* is a polyphagous pathogen and there is no evidence of host specificity, the existence of interactions between plant cultivars (e.g., soybean) and *M. phaseolina* genotypes aggressiveness has been demonstrated (Reznikov et al., 2018). Therefore, understanding the basis of the pathogenicity mechanisms as well as its interactions with host plants is crucial for controlling the pathogen.

## Host Plant - *M. Phaseolina* Interaction

In order to better understand the underlying mechanisms of resistance, several functional genomic strategies, including proteomics and transcriptomics, have been performed to analyse the interactions between several cultivars of various host plants and *M. phaseolina*. Hosts defense-related genes and proteins expressed during soilborne infection have been identified and huge datasets have been accumulated (Table 1).

**TABLE 1:** Study of the interactions between several host plants and *Macrophomina phaseolina.*

Host Plant	Study	Tools For Study	Results	References
Sorghum	Susceptible and resistant cultivars.	Gene expression analysis	Induction of chitinase and stilbene synthase genes	Sharma et al., 2014
Groundnut	Genotypes screening for disease tolerance.	Gene expression analysis	Induction of chitinase and β-1,3-glucanase genes	Iwuala et al., 2020
Jute	Evaluation of resistance level in a recombinant inbred line (RIL) population.	Transcriptomic profile and miRNA analysis	Induction of SA/MeJA1/ABA pathway genes	Biswas et al., 2014
	Identification of known and novel microRNAs in resistant RIL line.	*In silico* analysis	Nine novel microRNAs identified. Known microRNAs viz. miR-845b and miR-166 superfamily were abundantly expressed, and provide NBS-LRR and ROS mediated defense.	Dey et al., 2016
*Medicago truncatula*	Host-pathogen interaction at the molecular level. Treatment with methyl jasmonate (MJ) or ethylene (ET).	Gene expression analysis	Genes involved in flavonoid and isoflavonoid biosynthesis were up-regulated in the shoot. Genes in jasmonates (JAs) or ethylene (ET) pathways were not strongly induced in infected root tissue. Treatment with MJ or ET induced partial resistance.	Gaige et al., 2010
	Global gene expression profile at initial entry and colonization stages.	Transcriptomic profile	Regulation of genes involved in jasmonic acid and ethylene pathways. Regulation of genes involved in auxin homeostasis, polar auxin transport and auxin signalling. Treatment with exogenous auxin conferred partial resistance.	Mah et al., 2012
*Arabidopsis thaliana*	Defense response	Growth parameters. Gene expression analysis.	Reduction in shoot length, root length, photosynthetic pigments, relative water content and increase in sugar and proline contents in leaves. The expression of mitogen-activated protein kinases and thaumatin proteins increased while chitinase and beta-1,3-glucanase showed little increase compared with control plants.	Saima and Wu, 2019
	Semi-*in vitro* assay system to study Arabidopsis/*M. phaseolina* interaction	Transcriptomic profile	ET or JA mutants showed an enhanced susceptibility to *M. phaseolina*.	Schroeder et al., 2019
Potato	Evaluation of transgenic potato plants overexpressing Thaumatin-like proteins (TLPs) gene of Camellia sinensis (CsTLP).	Gene expression analysis.	Increase in transcripts of StPAL, StLOX, and StTLP genes involved in phenylpropanoid, lipoxygenase, and general defense response pathway.	Acharya et al., 2013
Soybean	Evaluation of susceptible (S) or moderately resistant (MR) genotypes under irrigated and nonirrigated and under fungal infested and noninfested conditions.	Analysis of total phenolics, lignins, total and cell wall boron and isoflavones in seed.	Significantly higher levels of phenolics, seed coat lignin, isoflavones, sugars, and total boron were observed in MR genotype than in S genotype seeds under irrigated and nonirrigated and under experimental *M. phaseolina* infested and noninfested conditions, indicating a possible association of these substances with resistance to toxin-mediated infection.	Bellaloui, 2012
	Genetic architecture of resistance and identification of causal genes.	Genome-wide association studies (GWAS).	Five and eight loci were reported for field and greenhouse screening, respectively, which were associated with candidate genes involved in controlling the plant defense response. No overlap of markers or genes was observed between field and greenhouse screenings.	Coser et al., 2017
	Defense response under *in vitro* conditions	Transcriptomic profile.	Induction of in secondary metabolism, hormone metabolism, stress, and signaling related genes.	Marquez et al., 2018
	Transgenic soybean with suppressed synthesis of isoflavones.	Molecular and biochemical characterization.	Reduced root capacity to produce glyceollin and increased susceptibility to pathogen infection.	Lygin et al., 2013

The interaction between two sorghum cultivars and *M. phaseolina* during the first hours of infection, resulted in the ovexpression of antifungal genes (i.e., chitinase and stilbene synthase), suggesting their roles in enhancing sorghum resistance (Sharma et al., 2014). Similarly, an increasing expression of chitinase and β-1,3-glucanase was noticed in groundnuts genotypes selected for their tolerance to *M. phaseolina* (Iwuala et al., 2020).

In the case of jute, a recombinant inbred line (RIL) population was studied via transcriptome and microRNA analysis. Defense genes related to the phenylpropanoid metabolism, phytohormones [jasmonic acid (JA), abscissic acid (ABA), ethylene (ET) and salycilic acid (SA)], signaling, cell wall biosynthesis and proteolysis were identified in this study (Biswas et al., 2014). Furthermore, microRNA analysis revealed highly abundant 22-nt miRNA families which have an innate ability to trigger phased small RNA cascades in SA/JA/ABA mediated natural SAR resistance (Biswas et al., 2014). Moreover, in-silico analysis suggested that a multi-layered defense is initiated by microRNAs to build strong barriers against *M. phaseolina* mediated by nucleotide binding site (NBS) and leucine-rich repeat (LRR) motifs, and the gene regulation of reactive oxygen species (ROS) (Dey et al., 2016).

*Medicago truncatula*, the main legume model, has also been used to analyze gene expression profile in response to *M. phaseolina* infection. This plant infected with *M. phaseolina* showed disease symptoms such as wilting and leaf yellowing at 1 day-post- inoculation (dpi), and most plants died 4 dpi. The expression of genes related to flavonoid and isoflavonoid biosynthesis, JA and ET pathways were induced. Meanwhile, transcriptome profile showed overall repression of auxin response genes. These results suggested that the host susceptibility to *M. phaseolina* is possibly partially due to suppression of the auxin response by the pathogen. In addition, plants treated with the active auxin, indole- 3-acetic acid (IAA), have been reported to be more tolerant to *M. phaseolina* (Gaige et al., 2010; Mah et al., 2012). On the other hand, studies in the model plant *Arabidopsis thaliana*, showed that increased expression of defense related genes, as mitogen-activated protein kinases and thaumatin proteins, with increased sugar and proline may play a role in the development of resistance against *M. phaseolina* (Saima and Wu, 2019). Additionally, ET or JA mutants showed an enhanced susceptibility to *M. phaseolina*. These observations suggested that ET and JA signaling pathways are important for protection against *M. phaseolina* in Arabidopsis (Schroeder et al., 2019).

The constitutive expression of *Camellia sinensis* thaumatin-like protein gene in potato confered enhanced resistance to *M. phaseolina* and *Phytophthora infestans* and showed a concomitant and significant increase in transcripts of LOX and phenylpropanoid pathways genes (Acharya et al., 2013).

Soybean is a leading agronomic crop with expanding production areas in diverse regions around the world. Charcoal rot caused by *M. phaseolina* is one of the most economically important soybean diseases (Wrather et al., 2010). This probably makes the interaction between soybean and *M. phaseolina* the more explored among host plants.

In the early 80s, Pearson et al. (1987) were the first to search for resistant soybean cultivars. Although this has not been succesfull to date, many studies have since been directed towards identifying new sources of resistance or even towards a better understanding of the resistance mechanisms that will contribute to future breeding programs (Bellaloui, 2012; Coser et al., 2017; Mengistu et al., 2018; Reznikov et al., 2018). Considering that the disease caused by *M. phaseolina* is highly correlated with environmental conditions, de Sousa Linhares et al. (2020) suggested the use of different temperatures for better characterization of the resistance levels, allowing the selection of plant cultivars most appropriated for different climatic conditions. Likewise, Mengistu et al. (2018) determined the severity of *M. phaseolina* in putative drough tolerante genotypes and determined the effect of charcoal rot on yield in irrigated and non-irrigated environments. Although a minimal relationship between charcoal rot disease severity and drought tolerance was observed, they concluded that it may be necessary to select for resistance to both traits in environments where both soil moisture stress and charcoal rot are high. The effect of charcoal rot infection was evaluated on seed total phenol, lignin, and isoflavone concentrations in soybean genotypes differing in their resistance to the disease under varying infestation levels and drought conditions. Results showed that resistance to charcoal rot have been associated with high levels of phenolic compounds, boron, and sugars in seeds (Bellaloui et al., 2012). Moreover, Genome-wide association studies (GWAS) provided useful information for understanding the genetic mechanisms of resistance and the advance of breeding programs, although no overlap of markers or genes have been observed between field and greenhouse experiments (Coser et al., 2017).

Transcriptome profile demonstrated a significant impact of *M. phaseolina* infection on soybean gene expression, including numerous plant defense genes related to signaling hormones, PR proteins, disease-resistance proteins, transcription factors, and secondary metabolism related genes (Marquez et al., 2018). Among secondary metabolism, phenylpropanoids (for example phytoalexins) are known to be involved in plant-pathogen interactions and can be strongly toxic or inhibitory to pathogens. Transgenic soybean lines with a gene that suppresses glyceollin (the collective name for soybean phytoalexins) biosynthesis were used to measure the effect of *M. phaseolina* colonization. These transformed soybeans markedly reduced root capacity to produce glyceollin and increased susceptibility to pathogen infection, showing that glyceollin accumulation is an important component of the innate soybean defense response (Lygin et al., 2013).

## Management Strategies

**FIGURE 4:**
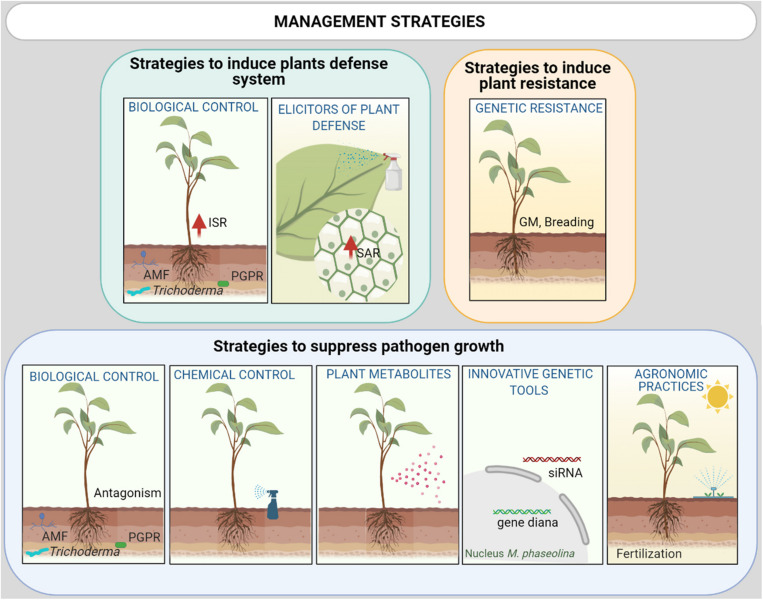
Integrated *M. phaseolina* disease management strategies could include a combination of: (i) Promotion of plant defense response with selected Biological control agents (BCAs) and natural or chemical elicitors via induced systemic resistance (ISR) or systemic acquired resistance (SAR), respectively; (ii) Host genetic resistance [via breeding or GM (genetic modification)]; (iii) Reduction of the inoculum density and growth via agricultural practices (biosolarization, irrigation), plant metabolites with allelopathic activity, BCAs, innovative genetic tools as Small interfering RNA (siRNA) molecules and chemical control using nanoformulation of fungicide with low collateral damage to surrounding ecosystems.

Many control strategies have been evaluated in recent decades with varying degrees of success (Figure 4). They are detailed in the section below (see Table 2).

**TABLE 2:** Summary of different management strategies against *Macrophomina phaseolina*.

Management Strategies	Host Plant	Disease	Experiment Condition	Type	References
*1. Genetic resistance*					
	Soybean	Charcoal Rot	Pot / Field experiment	Genotypic analysis, Histopathology, QTL mapping	Reznikov et al., 2018; Hemmati et al., 2018; da Silva et al., 2019
	Strawberry	Charcoal Rot	Pot experiment	Cultivar evaluation	Gomez et al., 2020
	Sesame	Charcoal Rot	*In vitro*	Cultivar evaluation	Chowdhury et al., 2014
	Sorghum	Stalk rot	In silico / Pot experiment	QTL mapping	Srinivasa Reddy et al., 2007; Mahmoud et al., 2018
	Cowpea	Damping-off / ashy stem blight	Pot / Field experiment	QTL mapping	Muchero et al., 2011
	Castor	Charcoal Rot	Field experiment	QTL mapping	Tomar et al., 2017
*2. Chemical control*					
	Soybean	Charcoal Rot	*In vitro* / Field experiment	Fungicide	Tonin et al., 2013; Reznikov et al., 2016
	Strawberry	Charcoal Rot	Field experiment	Fungicide	Chamorro et al., 2015a
	Green gram and black gram	Root Rot	In vitro / Pot experiment	Fungicide	Iqbal and Mukhtar, 2020
			In vitro	Fungicide	Cohen et al., 2012
			In vitro	Fungicide	Parmar et al., 2017
			In vitro	Fungicide	Lokesh et al., 2020
			In vitro	Fungicide	Swamy et al., 2018
			In vitro	Nanofungicide	Kumar et al., 2016
*3. Agronomic practices*					
	Strawberry	Charcoal Rot	Field experiment	Biosolarization	Chamorro et al., 2015b
	Soybean	Charcoal Rot	Field experiment	Irrigation	Kendig et al., 2000
	Soybean	Charcoal Rot	Field experiment	Crop Rotation	Francl et al., 1988
	Soybean	Charcoal Rot	Field experiment	Tillage system	Perez-Brandán et al., 2012
	Soybean	Charcoal Rot	Pot experiment	Fertilization	Spagnoletti et al., 2018; Spagnoletti et al., 2020
			Field experiment	Irrigation / Soil amendment	Lodha et al., 1997
	Soybean / Sunflower	Charcoal Rot	Pot experiment	Irrigation	Jordaan et al., 2019
*4. Biological control*					
*4.1 Fungi*					
	Groundnut	Charcoal Rot	Pot experiment	AMF	Doley and Jite, 2013
	Cowpea	Charcoal Rot	Pot experiment	AMF	Oyewole et al., 2017
	Soybean	Charcoal Rot	In vitro / Pot experiment	AMF	Marquez et al., 2018; Spagnoletti et al., 2017; Spagnoletti et al., 2020
	Sunflower	Charcoal Rot		AMF + PGPY	Nafady et al., 2019
			In vitro	*Trichoderma spp.*	Gajera et al., 2012
			In vitro	*Trichoderma spp.*	Sridharan et al., 2020
*4.2 Bacteria*					
	Chickpea	Charcoal Rot	Field experiment	*Pseudomonas sp.*	Kumar et al., 2007
	Flowering dogwood	Root Rot	Pot experiment	*Stenotrophomonas sp. Serratia sp.*	Mmbaga et al., 2018
	Groundnut	Charcoal Rot	In vitro / Pot experiment	*Bacillus spp.*	Sanjeevkumar et al., 2020
	Soybean	Charcoal Rot	In vitro / Pot experiment	*Pantoea sp. Pseudomonas sp. Bacillus spp.*	Vasebi et al., 2013; Simonetti et al., 2015
			In vitro	*Bacillus sp.*	Hussain and Khan, 2020
			In vitro	*Bacillus sp.*	Torres et al., 2016
			In vitro	*Burkholderia sp.*	Zaman et al., 2020
4.3 Mycovirus*					
*5. Plant metabolites*					
	Soybean	Charcoal Rot	Pot experiment	*Rosmarinus*	Lorenzetti et al., 2018
			In vitro	*Prosopis sp. Anacardium sp.*	Elaigwu et al., 2018
			In vitro	*Nigella sp.*	Aftab et al., 2019
			In vitro	*Mentha sp. Eucalyptus spp. Copaifera sp. Lippia sp.*	Ugulino et al., 2018
*6. Elicitors of plant defense*					
	Soybean	Charcoal Rot	Pot / Field experiment	Benzothiadiazole, Chitosan, Phenylalanine, Salicylic acid	Pawlowski et al., 2016
			In vitro	Chitosan	Chatterjee et al., 2014
*7. Innovative genetic tools*					
			In vitro	siRNAs	Forster and Shuai, 2020a; Forster and Shuai, 2020b

*None known mycoviruses are responsible for debilitation/hypovirulence of *M. phaseolina* or at least has been demonstrated.

### Genetic Resistance

To the best of our knowledge, there is no known vertical resistance (R-gene based) to *M. phaseolina* inhibiting or limiting infection but rather, a partial resistance which do not limit infection but reduce or compensate the damages, and therefore the consequences on the fitness of plants.

Cultivars of soybean and strawberry with varying degrees of resistance to *M. phaseolina* have been identified (Reznikov et al., 2018; Gomez et al., 2020). Differences in fungal behaviour close to the roots and during infection of roots have been observed between resistant vs. susceptible varieties of sesame. The rhizosphere around the resistant variety had a reduced growth of *M. phaseolina* as compared to the susceptible variety (Chowdhury et al., 2014). Similarly, Hemmati et al. (2018) reported the formation of adventitious roots around the crown of soybean and inability of the pathogen to complete its life cycle in resistant cultivars, while pre-penetration steps within the roots were not linked to resistance, as they did not observe differences in microsclerotia germination and hyphae development.

Notably, the identification and mapping of QTLs associated with resistance to *M. phaseolina*, revealed candidate genes with potential for further functional genomics analysis and it may facilitate breeding and molecular engineering progress against this pathogen (Srinivasa Reddy et al., 2007; Muchero et al., 2011; Tomar et al., 2017; Mahmoud et al., 2018; da Silva et al., 2019).

### Chemical Control

The chemical control of *M. phaseolina* is difficult, since there are no systemic fungicides that move towards the root. As far as we know, no fungicides have been registered to control this pathogen. However, systemic and non-systemic fungicides (i.e., carbendazim, difenoconazole, benomyl, azoxystrobin, dazome) at different concentration were evaluated *in vitro* and *in vivo* against *M. phaseolina* (Cohen et al., 2012; Tonin et al., 2013; Chamorro et al., 2015a; Parmar et al., 2017; Lokesh et al., 2020).

Results indicates that the mycelial growth and formation of sclerotia are highly sensitive to carbendazim (50 ppm), an impact that increases with the increase in concentration of this systemic fungicide (Lokesh et al., 2020). Carbendazim inactivates tubulin function, the building block of microtubules, necessary for the fungal growth (Davidse and Flach, 1978). In addition, in another set of experiments, carbendazim application reduced disease incidence and increased the rate of plant survival (Iqbal and Mukhtar, 2020). Interestingly, the nanoformulation (particle size < 100 nm) of the commercial fungicide Trifloxystrobin 25% + Tebuconazole 50% (75 WG), was better in comparison to the conventional one (micro sized). The nanoform was effective at 10 ppm and it exerted hyphal abnormality, hyphal lysis and abnormality of sclerotial formation on *M. phaseolina* when tested under *in vitro* conditions (Kumar et al., 2016).

Disease management combining cultural practices with chemicals have been reported, but no conclusive results could be drawn, requiring further investigations (Cohen et al., 2012). Although the efficacy of certain chemical fumigants has been demonstrated (Iqbal and Mukhtar, 2020; Lokesh et al., 2020), agro-environmental policies and the increasing negative perception of the public on the agrochemicals have led to the evaluation and comparison of chemicals agents with more sustainable alternatives to control plant diseases caused by *M. phaseolina* (Reznikov et al., 2016; Swamy et al., 2018; Adhikary et al., 2019).

### Agronomic Practices

There is a relationship between pathogen inoculum density in soil and disease intensity, and between disease intensity and yield loss. Hence, some agricultural practices have intended to reduce the inoculum density. Biosolarization, a technique that combines biofumigation and solarization, has been shown effective in the reduction or stabilization of *M. phaseolina* microsclerotia population in soil, reducing the incidence of strawberry charcoal rot (Chamorro et al., 2015b). Conversely, irrigation maintained densities of microsclerotia relatively constant and did not prevent infection by *M. phaseolina*. However, high soil moisture (above 60%) reduced disease severity (Kendig et al., 2000; Jordaan et al., 2019). The wide host range and high persistence of *M. phaseolina* microsclerotia make crop rotation, intercropping and lay period strategies less considered. Although crop rotation has not been effective in controlling this pathogen, reduced densities of inoculum occurred when soybean was less frequently used in rotations (Francl et al., 1988). For the particular case of sesame, grown as mixed or inter cropped with green gram, less incidence of *Macrophomina* stem and root rot and higher seed yield equivalent as compared to sole sesame was observed (Rajpurohit, 2002).

Approaches intended to modify the soil environment, favouring antagonistic organisms interfering with the pathogen, have also been attempted. For example, the adoption of conservation strategies as direct seeding, showed a suppression of *M. phaseolina* favoured by the higher microbial abundance and activity, and the subsequent development of plants with healthier root systems (Perez-Brandán et al., 2012). Similarly, combining irrigation with soil amendment, increased the population of lytic bacteria against *M. phaseolina* (Lodha et al., 1997). Finally, fertilization has shown different effects on the severity of *M. phaseolina.* Phosphorus fertilization have shown a reduction, while nitrogen increased disease severity (Spagnoletti et al., 2018, 2020).

### Biological Control

Biological control agents (BCAs) has well as plant metabolites and elicitors of plant defenses have received increasing attention in the last few decades. Some BCAs impact the pathogens directly, inhibiting their growth, while others affect the pathogen indirectly by eliciting defense pathways in the host plant.

#### Fungal BCAs

Arbuscular mycorrhizal fungi (AMF) are probably the oldest and most widespread symbiosis on earth (Smith and Carvil, 1997) forming mutualistic associations with an estimate of 72% of land plants (Brundrett and Tedersoo, 2018). They produce significant changes in the host plant and its environment and have been repeatedly reported to reduce the incidence or severity of several pests and diseases (Pozo et al., 2010; Eke et al., 2016; Karthikeyan et al., 2016; Zhang et al., 2018; Jain and Pundir, 2019; Marquez et al., 2019). Described mechanisms range from competition with soil-borne pathogens for space and nutrients to reprogramming of plant gene expression and metabolism, particularly those involved to defense mechanisms (Liu et al., 2003, 2007; López-Ráez et al., 2010; Campos-Soriano et al., 2012; Gallou et al., 2012; Rivero et al., 2015; Marquez et al., 2018, 2019). These fungi may also prime host tissues for efficient activation of plant defenses upon a challenger attack (Pozo and Azcón-Aguilar, 2007).

Although mycorrhizal symbiosis is not able to avoid infection *M. phaseolina* or any other pathogens, a reduction in root infection by *M. phaseolina* and disease symptoms severity have been reported (Doley and Jite, 2013; Oyewole et al., 2017; Spagnoletti et al., 2017, 2020; Marquez et al., 2018). These observations were associated with a decreased oxidative damage and the boosting of defense response mechanisms, including a significant increase in total phenol and proline contents, and superoxidase activity (Doley and Jite, 2013; Spagnoletti et al., 2017). In addition, transcriptional studies have suggested that protection is associated with the modulation of pathogen infection. The induction of serine carboxipeptidase-like (SCPL) and lectin genes have been proposed to enhance pathogen recognition capacity, allowing an early defense response, a lower incidence of disease, and better cell homeostasis in roots. However, it is important to notice that 40% of the genes differentially expressed, in mycorrhizal soybean plants infected with *M. phaseolina*, and potentially involved in the defense response of the plant, corresponded to unknown genes or genes without assigned function (Marquez et al., 2018). Further studies on these interactions should be conducted for a better understanding of the mechanisms involved in the biological control mediated by AMF. Likewise, to the best of our knowledge, the effect of AMF on the pathogenicity mechanisms of *M. phaseolina* has not yet been investigated. Eventually, even though phosphorus fertilization have shown a reduced disease severity of *M. phaseolina*, a partial reduction of the AMF protection was observed when both combined treatments where applied (Spagnoletti et al., 2018). Conversely, AMF protects soybean plants against *M. phaseolina* even under nitrogen fertilization (Spagnoletti et al., 2020). Hence, its important to consider the effect of integrated management in agricultural practices.

*Trichoderma spp.* are effective BCAs for several soilborne fungal plant pathogens including *M. phaseolina* (Bastakoti et al., 2017; Hyder et al., 2017). These saprotrophic fungi have evolved multiple antagonistic mechanisms such as nutrient competition, antibiotic production, and mycoparasitism. Moreover, some species are known for their effects on plant health, such as plant growth promotion effects or the abilities to enhance systemic resistance (Martinez-Medina et al., 2016).

*M. phaseolina* growth inhibitions during antagonism was positively correlated with the capacity of *Trichoderma spp.* to overgrowth and degrade the pathogen mycelia (coiling around the hyphae with apressoria and hook-like structure). The induction of chitinase, β-1, 3 glucanase and increase in total phenol content was also observed, suggesting their role in growth inhibition of pathogen during antagonism (Gajera et al., 2012). Similarly, *Brettanomyces naardensis*, an antagonistic and growth-promoting yeast, is a potent biocontrol agent *for M. phaseolina* that colonizes fungal hyphae causing malformation and damage (Nafady et al., 2019).

In addition to inhibiting the growth of the pathogen during direct interaction, the antibiosis via microbial volatile organic compounds (mVOCs) was observed in the case of *Trichoderma longibrachiatum* (Sridharan et al., 2020). These mVOCs reduced *M. phaseolina* mycelial growth by altering the mycelial structure. Interactions increased the level of terpenoids, which includes longifolene, caryophyllene, and cuprenene, but also resulted in newly expressed compound, which were not produced by none of the organisms before interaction, as limonene, azulene, 3-methyl-1-butanol, styrene, salicylaldehyde, undecane, and 3-methylphenol. These compounds might act as signaling molecules in microbe-microbe interactions and are potent antimicrobials.

#### Bacterial BCAs

Several rhizospheric and root-associated bacteria have been isolated and tested for their antagonistic effects against *M. phaseolina*. Several isolates were quite effective in reducing disease incidence and promoting host plant growth traits. Among these are species belonging to *Bacillus*, *Pantoea*, *Pseudomonas*, *Stenotrophomonas*, and *Serratia* genus (Kumar et al., 2007; Vasebi et al., 2013; Torres et al., 2016; Mmbaga et al., 2018; Sanjeevkumar et al., 2020; Hussain and Khan, 2020).

*Bacillus* isolates have shown important inhibitions of *Macrophomina* sp. growth, either in dual culture plate tests or through the use of culture filtrates. *Bacillus amyloliquefaciens* and *B. siamensis* have shown antifungal activities via the excretion of compounds of the lipopeptides-surfactin class, although further studies are required to understand the exact composition and molecular structure of the filtrates. For instance, a lethal damage on the fungus microsclerotia was observed (Torres et al., 2016; Hussain and Khan, 2020). Meanwhile, *B. subtilis* generated a fungistatic effect probably connected to a competition for space or nutrients, instead of a toxic effect (Torres et al., 2016). Furthermore, two plant growth promoting rhizobacteria (PGPR) isolates, identified as *Pseudomonas fluorescens* and *Bacillus subtilis*, have shown inhibitory capacities against *M. phaseolina* under *in vitro* (on soybean seeds) and *in vivo* (greenhouse assay) culture conditions (Simonetti et al., 2015).

Interestingly, the whole proteome of *M. phaseolina* upon *B. contaminans* challenged condition identified the upregulatation of proteins related to energy production and defense and stress response, while there was significant downregulation in oxidative stress protection pathways, growth and cell wall integrity, and virulence. *M. phaseolina* remained dormant while it revert to an active life with reduced virulence once the bacteria was removed. In this regard, it seems that *B. contaminans* arrest the growth of *M. phaseolina* and decrease its pathogenicity (Zaman et al., 2020).

#### Mycoviral BCAs

In nature, some mycoviruses are known to be responsible for debilitation/hypovirulence of plant pathogens (Xie and Jiang, 2014). Although several virus-infecting *M. phaseolina* isolates were described (Wang et al., 2019a, b), no hypovirulence has been documented of this pathogenic fungus or at least was demonstrated.

### Plant Metabolites

Most plants exhibit inhibitory and stimulatory biochemical interactions with other plants and microorganisms, referred to as “allelopathy.” Especially, through root exudates, higher plants are able to prevent phytopathogens from infecting crops (Ushiki et al., 1996). Plant extracts and their volatile oils have been reported as natural phytosanitary products aiming the substitution or reduction in the application of conventional fungicides.

In plant defense systems, secondary metabolites can be divided into distinct chemical groups: terpenes, phenolics, nitrogen and sulfur containing compounds. A high number of secondary metabolites possesses antifungal characteristics (Zaynab et al., 2018).

Whole plant or leaf extracts of medicinal plants viz: *Prosopis africana*, *Anacardium occidentale* and *Nigella sativa* have been assayed against *M. phaseolina*, observing an inhibition of its growth. Analysis of the extracts showed the presence of alkaloids, saponins, tannins, flavonoids, anthraquinones, octadecadienoic acid, pentadecanoic acid, 1,2,3,4, butaneteterol, octadecanoic acid and linoleic acid. The antifugal activity of these extracts have been confirmed in several studies (Elaigwu et al., 2018; Aftab et al., 2019). Moreover, some extracts were able to induce the activity of defense enzymes in soybean plants inoculated with *M. phaseolina* (Lorenzetti et al., 2018). Additionally, *Lippia gracilis* oil extract showed an important inhibitory effect on the mycelial growth of *M. phaseolina*, becoming a promising alternative as control method (Ugulino et al., 2018). Furthermore, exogenous application of the synthetic strigolactone (SL) GR24 suppressed *M. phaseolina* hyphal branching. These results suggests that SLs released by plant roots, not only affect AMF and parasitic plants, but they also may play other important roles by affecting other organisms in the plant environment (Dor et al., 2011).

### Elicitors of Plant Defense

Elicitors are natural or synthetic compounds, which sprayed on the plants have been shown to induce systemic acquired resistance (SAR) and deter infection from bacterial, fungal, and viral pathogens. In order to control *M. phaseolina* and two other soybean pathogens (*Phytophtora sojae* and *Sclerotinia sclerotiorum*), the elicitors benzothiadiazole (BTH), chitosan (CHT), phenylalanine (PHE), and salicylic acid (SA), have been applied to soybean foliage. Results showed that the elicitor effectiveness varied based on soybean genotypes, pathogens, and environmental conditions (Pawlowski et al., 2016).

Chitosan has shown a potential dual role by inducing defense response in jute seedlings and directly inhibiting *M. phaseolina* during infection. Changes in enzyme profiles of jute after treatment with water-soluble chitosan (s-chitosan) helped to understand the mode of action of this antifungal compound. In this sense, the activity of defense related enzymes like chitosanase and peroxidase in infected seedlings was observed to be enhanced after treatment with s-chitosan in jute seedlings during infection by *M. phaseolina* (Chatterjee et al., 2014).

A better understanding of the immune responses triggered by elicitors is necessary. The use of elicitors in plant resistance may be detrimental to other physiological processes impacting negatively other plant traits, such as biomass and seed production. Therefore, it is important to make distinction between elicitors that directly activate plant defenses and those which acts as priming compounds. Priming condition, whereby plants that have been subjected to prior stimulus will respond more quickly or more strongly to a subsequent attack, is thought to be a relatively low-cost mechanism of advancing plant defense (Paré et al., 2005; Conrath, 2011; Denancé et al., 2013).

### Innovative Genetic Tools

Small interfering RNA (siRNA) molecules have been used as a tool for the management of many plant pathogens (i.e., *Fusarium*, *Aspergillus*, *Verticillium, Sclerotinia*) (Mcloughlin et al., 2018). RNAi-mediated suppression of selected target genes, chosen based on their importance in growth and/or pathogenicity, can negatively affect the pathogen’s ability to infect the host or minimizing host symptoms.

Exogenous siRNAs were applied to target genes, β-1,3-glucan synthase and chitin synthase, in *M. phaseolina*. These targeting genes are important for the fungal cell wall synthesis. Interestingly, growth of siRNA-treated fungi has been suppressed, as indicated by smaller growth area and less dense mycelium. The siRNA treatments have also been reported to delay the maturation of the fungus since microsclerotia developed and melanized at a slower pace under multiple treatment conditions. Moreover, *M. phaseolina* growth suppression was correlated with a significant decreases in transcript abundances of target genes (Forster and Shuai, 2020a, b). Selection of siRNAs, where undesirable results due to off-target binding in a host plant or other organisms are minimized, is very important as they can be used for application in other innovative technologies. For example, Host-Delivered RNA interference (HD-RNAi), where plants contain genes encoding siRNA targeting toward pathogens (Hu et al., 2015).

## Concluding Remarks

The interactions that occur underground, between *M. phaseolina* and micro or macrooganisms and even with the physico-chemical environment conditions, are very complex and it is therefore of uppermost important to fully understand them to optimize their application in disease control strategies. Any management strategy should include interference, alteration, or manipulation of at least one of these components or the interactions, with minimal disruption to the environment and natural resources. Responsible management of diseases caused by *M. phaseolina* is essential, since the importance of this soilborne fungus lies not only in the losses it causes but also in the impacts it has on the environmental due to unsustainable management practices (Vimal et al., 2017).

## Author Contributions

NM, MG, SD, and DD contributed to conception and design of the manuscript. NM wrote the first draft of the manuscript and organized the tables. MG performed the figures. NM, MG, and SD contributed to manuscript revision, read, and approved the submitted version.

## Conflict of Interest

The authors declare that the research was conducted in the absence of any commercial or financial relationships that could be construed as a potential conflict of interest.

## References

[B1] Abbas H. K. Bellaloui N. Butler A. M. Nelson J. L. Abou-Karam M. Shier W. T. (2020). Phytotoxic responses of soybean (Glycine max L.) to Botryodiplodin, a Toxin produced by the charcoal rot disease fungus, *Macrophomina phaseolina*. *Toxins (Basel)* 12:25. 10.3390/toxins12010025 31906290 PMC7020515

[B2] Acharya K. Pal A. K. Gulati A. Kumar S. Singh A. K. Ahuja P. S. (2013). Overexpression of Camellia sinensis thaumatin-like protein, CsTLP in potato confers enhanced resistance to *macrophomina phaseolina* and phytophthora infestans infection. *Mol. Biotechnol.* 54 609–622. 10.1007/s12033-012-9603-y 23086453

[B3] Adhikary N. K. Chowdhury M. R. Begum T. Mallick R. (2019). Integrated management of stem and root rot of sesame (*Sesamum indicum* L.) caused by *Macrophomina phaseolina* (Tassi) Goid. *Int. J. Curr. Microbiol. Appl. Sci.* 8 804–808. 10.20546/ijcmas.2019.804.089

[B4] Aftab A. Yousaf Z. Javaid A. Riaz N. Younas A. Rashid M. (2019). Antifungal activity of vegetative Methanolic Extracts of Nigella sativa against Fusarium oxysporum and *Macrophomina phaseolina* and its Phytochemical Profiling by GC-MS analysis. *Intl. J.Agric. Biol.* 21 569–576. 10.17957/IJAB/15.0930 29653435

[B5] Almeida ÁM. R. Abdelnoor R. V. Arrabal Arias C. A. Carvalho V. P. Jacoud Filho S. D. Marin S. R. R. (2003). Genotypic diversity among brazilian isolates of *Macrophomina phaseolina* revealed by RAPD. *Fitopatol. Bras* 28 279–285.

[B6] Almomani F. Alhawatema M. Hameed K. (2013). Detection, identification and morphological characteristic of *Macrophomina phaseolina*: the charcoal rot disease pathogens isolated from infected plants in Northern Jordan. *Arch. Phytopathol. Plant Prot.* 46 1005–1014. 10.1080/03235408.2012.756174

[B7] Babu B. K. Mesapogu S. Sharma A. Somasani S. R. Arora D. K. (2011). Quantitative real-time PCR assay for rapid detection of plant and human pathogenic *Macrophomina phaseolina* from field and environmental samples. *Mycologia* 103 466–473. 10.3852/10-181 21186328

[B8] Babu B. K. Reddy S. S. Yadav M. K. Sukumar M. Mishra V. Saxena A. K. (2010). Genetic diversity of *Macrophomina phaseolina* isolates from certain agro-climatic regions of India by using RAPD markers. *Indian J. Microbiol.* 50 199–204. 10.1007/s12088-010-0033-x 23100828 PMC3450334

[B9] Babu B. K. Saxena A. K. Srivastava A. K. Arora D. K. (2007). Identification and detection of *Macrophomina phaseolina* by using species-specific oligonucleotide primers and probe. *Mycologia* 99 797–803. 10.3852/mycologia.99.6.797 18333503

[B10] Bandara Y. M. A. Y. Weerasooriya D. K. Liu S. Little C. R. (2018). The necrotrophic fungus *macrophomina phaseolina* promotes charcoal rot susceptibility in grain sorghum through induced host cell-wall-degrading enzymes. *Phytopathology* 108 948–956. 10.1094/PHYTO-12-17-0404-R 29465007

[B11] Bastakoti S. Belbase S. Manandhar S. Arjyal C. (2017). Trichoderma species as Biocontrol agent against soil borne fungal pathogens. *Nepal J. Biotechnol.* 5 39–45. 10.3126/njb.v5i1.18492

[B12] Bellaloui N. (2012). Soybean Seed Phenol, Lignin, and Isoflavones and sugars composition altered by foliar boron application in soybean under water stress. *Food Nutr. Sci.* 3 579–590. 10.4236/fns.2012.34080

[B13] Bellaloui N. Mengistu A. Zobiole L. H. S. Shier W. T. (2012). Resistance to toxin-mediated fungal infection: role of lignins, isoflavones, other seed phenolics, sugars, and boron in the mechanism of resistance to charcoal rot disease in soybean. *Toxin Rev.* 31 16–26. 10.3109/15569543.2012.691150

[B14] Bhattacharya G. Dhar T. K. Kathleen Bhattacharya F. Siddiqui K. A. (1987). Mutagenic action of phaseolinone, a Mycotoxin isolated from *Macrophomina phaseolina*. *Aust. J. Biol. Sci.* 40 349–353. 10.1071/BI9870349 3330928

[B15] Biswas C. Dey P. Karmakar P. G. Satpathy S. (2014). Next-generation sequencing and micro RNAs analysis reveal SA/MeJA1/ABA pathway genes mediated systemic acquired resistance (SAR) and its master regulation via production of phased, trans-acting siRNAs against stem rot pathogen *Macrophomina phaseolina* in a. *Physiol. Mol. Plant Pathol.* 87 76–85. 10.1016/j.pmpp.2014.07.003

[B16] Brundrett M. C. Tedersoo L. (2018). Evolutionary history of mycorrhizal symbioses and global host plant diversity. *New Phytol.* 220 1108–1115. 10.1111/nph.14976 29355963

[B17] Burkhardt A. K. Childs K. L. Wang J. Ramon M. L. Martin F. N. (2019). Assembly, annotation, and comparison of *Macrophomina phaseolina* isolates from strawberry and other hosts. *BMC Genomics* 20:802. 10.1186/s12864-019-6168-1 31684862 PMC6829926

[B18] Burkhardt A. Ramon M. L. Smith B. Koike S. T. Martin F. (2018). Development of molecular methods to detect *Macrophomina phaseolina* from strawberry plants and soil. *Phytopathology* 108 1386–1394. 10.1094/PHYTO-03-18-0071-R 29869955

[B19] Campos-Soriano L. García-Martinez J. Segundo B. S. (2012). The arbuscular mycorrhizal symbiosis promotes the systemic induction of regulatory defence-related genes in rice leaves and confers resistance to pathogen infection. *Mol. Plant Pathol.* 13 579–592. 10.1111/j.1364-3703.2011.00773.x 22212404 PMC6638712

[B20] Chamorro M. Domínguez P. Medina J. J. Miranda L. Soria C. Romero F. (2015a). Assessment of chemical and biosolarization treatments for the control of *Macrophomina phaseolina* in strawberries. *Sci. Hortic. (Amsterdam)* 192 361–368. 10.1016/j.scienta.2015.03.029

[B21] Chamorro M. Miranda L. Domínguez P. Medina J. J. Soria C. Romero F. (2015b). Evaluation of biosolarization for the control of charcoal rot disease (*Macrophomina phaseolina*) in strawberry. *Crop Prot.* 67 279–286. 10.1016/j.cropro.2014.10.021

[B22] Chatterjee S. Chatterjee B. P. Guha A. K. (2014). International journal of biological macromolecules a study on antifungal activity of water-soluble chitosan against *Macrophomina phaseolina*. *Int. J. Biol. Macromol.* 67 452–457. 10.1016/j.ijbiomac.2014.04.008 24747381

[B23] Chowdhury S. Basu A. Ray Chaudhuri T. Kundu S. (2014). In-vitro characterization of the behaviour of *Macrophomina phaseolina* (Tassi) Goid at the rhizosphere and during early infection of roots of resistant and susceptible varieties of sesame. *Eur. J. Plant Pathol.* 138 361–375. 10.1007/s10658-013-0335-z

[B24] Cohen R. Omari N. Porat A. Edelstein M. (2012). Management of *Macrophomina* wilt in melons using grafting or fungicide soil application: pathological, horticultural and economical aspects. *Crop Prot.* 35 58–63. 10.1016/j.cropro.2011.12.015

[B25] Conrath U. (2011). Molecular aspects of defence priming. *Trends Plant Sci.* 16 524–531. 10.1016/j.tplants.2011.06.004 21782492

[B26] Coser S. M. Chowda Reddy R. V. Zhang J. Mueller D. S. Mengistu A. Wise K. A. (2017). Genetic architecture of charcoal rot (*Macrophomina phaseolina*) resistance in soybean revealed using a diverse panel. *Front. Plant Sci.* 8:1626. 10.3389/fpls.2017.01626 28983305 PMC5613161

[B27] Crous P. W. Slippers B. Wingfield M. J. Rheeder J. Marasas W. F. O. Philips A. J. L. (2006). Phylogenetic lineages in the Botryosphaeriaceae. *Stud. Mycol.* 55 235–253. 10.3114/sim.55.1.235 18490983 PMC2104729

[B28] da Silva M. P. Klepadlo M. Gbur E. E. Pereira A. Mason R. E. Rupe J. C. (2019). QTL mapping of charcoal rot resistance in PI 567562A soybean accession. *Crop Sci.* 59 474–479. 10.2135/cropsci2018.02.0145

[B29] Davidse L. C. Flach W. (1978). Interaction of thiabendazole with fungal tubulin. *Biochim. Biophys. Acta* 543 82–90. 361093 10.1016/0304-4165(78)90456-7

[B30] de Sousa Linhares C. M. Ambrósio M. M. Q. Castro G. Torres S. B. Esteras C. de Sousa Nunes G. H. (2020). Effect of temperature on disease severity of charcoal rot of melons caused by *Macrophomina phaseolina*: implications for selection of resistance sources. *Eur. J. Plant Pathol* 158 431–441. 10.1007/s10658-020-02083-w

[B31] Denancé N. Sánchez-Vallet A. Goffner D. Molina A. (2013). Disease resistance or growth: the role of plant hormones in balancing immune responses and fitness costs. *Front. Plant Sci.* 4:155. 10.3389/fpls.2013.00155 23745126 PMC3662895

[B32] Dey P. Biswas C. Karmakar P. G. (2016). Identification and characterization of differentially expressed novel miRNAs (21-24 nt) in a *Macrophomina phaseolina* resistant RIL line of jute (*Corchorus capsularis* L.). *Physiol. Mol. Plant Pathol.* 94 62–66. 10.1016/j.pmpp.2016.04.005

[B33] Dhingra O. D. Sinclair J. B. (1978). *Biology and Pathology of Macrophomina Phaseolina.* Minas Gerais: Universidade Federal de Viçosa: Impresa Universitaria.

[B34] Doley K. Jite P. K. (2013). Effect of arbuscular mycorrhizal fungi on growth of groundnut and disease caused by *Macrophomina phaseolina*. *J. Exp. Sci.* 2012 46–50.

[B35] Dor E. Joel D. M. Kapulnik Y. Koltai H. Hershenhorn J. (2011). The synthetic strigolactone GR24 influences the growth pattern of phytopathogenic fungi. *Planta* 234 419–427. 10.1007/s00425-011-1452-6 21688170

[B36] Eke P. Chatue Chatue G. Wakam L. N. Kouipou R. M. T. Fokou P. V. T. Boyom F. F. (2016). Mycorrhiza consortia suppress the fusarium root rot (*Fusarium solani* f. sp. Phaseoli) in common bean (*Phaseolus vulgaris* L.). *Biol. Control* 103 240–250. 10.1016/j.biocontrol.2016.10.001

[B37] Elaigwu M. Oluma H. O. A. Onekutu A. (2018). Phytochemical and antifungal activity of leaf extracts of Prosopis africana and Anacardium occidentale against *Macrophomina* Root Rot of *Sesamum indicum* L. in Benue State, Central Nigeria. *J. Geosci. Environ. Prot.* 6 66–76. 10.4236/gep.2018.67005

[B38] Forster H. Shuai B. (2020a). Exogenous siRNAs against chitin synthase gene suppress the growth of the pathogenic fungus *Macrophomina phaseolina*. *Mycologia* 112 699–710. 10.1080/00275514.2020.1753467 32615881

[B39] Forster H. Shuai B. (2020b). RNAi-mediated knockdown of β-1,3-glucan synthase suppresses growth of the phytopathogenic fungus *Macrophomina phaseolina*. *Physiol. Mol. Plant Pathol.* 110:101486. 10.1016/j.pmpp.2020.101486

[B40] Francl L. J. Wyllie T. D. Rosenbrock S. M. (1988). Influence of crop rotation on population density of *Macrophomina phaseolina* in soil infested with Heterodera glycines. *Plant Dis* 72 760–764.

[B41] Gaige A. R. Ayella A. Shuai B. (2010). Methyl jasmonate and ethylene induce partial resistance in *Medicago truncatula* against the charcoal rot pathogen *Macrophomina phaseolina*. *Physiol. Mol. Plant Pathol.* 74 412–418. 10.1016/j.pmpp.2010.07.001

[B42] Gajera H. Bambharolia R. Patel S. Khatrani T. Goalkiya B. (2012). Antagonism of *Trichoderma* spp. against *Macrophomina phaseolina*: evaluation of coiling and cell wall degrading enzymatic activities. *J. Plant Pathol. Microbiol.* 3:149. 10.4172/2157-7471.1000149

[B43] Gallou A. Declerck S. Cranenbrouck S. (2012). Transcriptional regulation of defence genes and involvement of the WRKY transcription factor in arbuscular mycorrhizal potato root colonization. *Funct. Integr. Genomics* 12 183–198. 10.1007/s10142-011-0241-4 21811781

[B44] Ghosh T. Biswas M. K. Guin C. Roy P. (2018). A review on characterization, therapeutic approaches and pathogenesis of *Macrophomina phaseolina*. *Plant Cell Biotechnol. Mol. Biol.* 19 72–84.

[B45] Gomez A. O. De Faveri J. Neal J. M. Aitken E. A. B. Herrington M. E. (2020). Response of strawberry cultivars inoculated with *Macrophomina phaseolina* in Australia. *Int. J. Fruit Sci.* 20 1–14. 10.1080/15538362.2019.1709114

[B46] Gupta G. K. Sharma S. K. Ramteke R. (2012). Biology, epidemiology and management of the pathogenic fungus *Macrophomina phaseolina* (Tassi) goid with special reference to charcoal rot of soybean (Glycine max (L.) Merrill). *J. Phytopathol.* 160 167–180. 10.1111/j.1439-0434.2012.01884.x

[B47] Hemmati P. Zafari D. Mahmoodi S. B. Hashemi M. Gholamhoseini M. Dolatabadian A. (2018). Histopathology of charcoal rot disease (*Macrophomina phaseolina*) in resistant and susceptible cultivars of soybean. *Rhizosphere* 7 27–34. 10.1016/j.rhisph.2018.06.009

[B48] Hu Z. Parekh U. Maruta N. Trusov Y. Botella J. R. (2015). Down-regulation of *Fusarium oxysporum* endogenous genes by Host-Delivered RNA interference enhances disease resistance. *Front. Chem.* 3:1. 10.3389/fchem.2015.00001 25654075 PMC4299518

[B49] Hussain T. Khan A. A. (2020). Determining the antifungal activity and characterization of *Bacillus siamensis AMU03* against *Macrophomina phaseolina* (Tassi) Goid. *Indian Phytopathol.* 73 507–516. 10.1007/s42360-020-00239-6

[B50] Hyder S. Inam-ul-haq M. Bibi S. Humayun A. (2017). Novel potential of *Trichoderma* Spp. as biocontrol agent. *J. Entomol. Zool. Stud.* 5 214–222.

[B51] Iqbal U. Mukhtar T. (2020). Inhibitory effects of some fungicides against *Macrophomina phaseolina* causing charcoal rot. *Pak. J. Zool.* 52 709–715. 10.17582/journal.pjz/20181228101230

[B52] Islam M. Haque M. Islam M. Emdad E. Halim A. Hossen Q. M. (2012). Tools to kill: genome of one of the most destructive plant pathogenic fungi *Macrophomina phaseolina*. *BMC Genomics* 13:493. 10.1186/1471-2164-13-493 22992219 PMC3477038

[B53] Iwuala E. Odjegba V. Unung O. Alam A. (2020). Expression of stress responsive β-1,3-glucanase and chitinase genes in Arachis hypogaea seedlings against *Macrophomina phaseolina*. *Gene Rep.* 20:100693. 10.1016/j.genrep.2020.100693

[B54] Jain P. Pundir R. K. (2019). “Biocontrol of soil phytopathogens by arbuscular mycorrhiza – a review,” in *Mycorrhizosphere and Pedogenesis*, eds Varma A. Choudhary D. (Singapore: Springer). 10.1007/978-981-13-6480-8_14

[B55] Jana T. K. Singh N. K. Koundal K. R. Sharma T. R. (2005). Genetic differentiation of charcoal rot pathogen, *Macrophomina phaseolina*, into specific groups using URP-PCR. *Can. J. Microbiol.* 51 159–164. 10.1139/w04-122 16091774

[B56] Jordaan E. van der Waals J. E. McLaren N. W. (2019). Effect of irrigation on charcoal rot severity, yield loss and colonization of soybean and sunflower. *Crop Prot.* 122 63–69. 10.1016/j.cropro.2019.04.026

[B57] Karthikeyan B. Abitha B. Henry A. J. Sa T. Joe M. M. (2016). “Interaction of Rhizobacteria with Arbuscular Mycorrhizal fungi (AMF) and their role in stress abetment in agriculture,” in *Recent Advances on Mycorrhizal Fungi*, ed. Pagano M. C. (Cham: Springer), 117–142. 10.1007/978-3-319-24355-9

[B58] Kaur S. Dhillon G. S. Brar S. K. Vallad G. E. Chand R. Chauhan V. B. (2012). Biology, economic importance and current diagnostic trends. *Crit. Rev. Microbiol.* 38 136–151. 10.3109/1040841X.2011.640977 22257260

[B59] Kendig S. R. Rupe J. C. Scott H. D. (2000). Effect of irrigation and soil water stress on densities of *Macrophomina phaseolina* in soil and roots of two soybean cultivars. *Plant Dis.* 84 895–900. 10.1094/PDIS.2000.84.8.895 30832145

[B60] Khan A. N. Shair F. Malik K. Hayat Z. Khan M. A. Hafeez F. Y. (2017). Molecular identification and genetic characterization of *Macrophomina phaseolina* strains causing pathogenicity on sunflower and chickpea. *Front. Microbiol.* 8:1309. 10.3389/fmicb.2017.01309 28769890 PMC5515817

[B61] Kumar D. G. Natarajan N. Nakkeeran S. (2016). Antifungal activity of nanofungicide Trifloxystrobin 25% + Tebuconazole 50% against *Macrophomina phaseolina*. *African J. Microbiol. Res.* 10 100–105. 10.5897/AJMR2015.7692

[B62] Kumar V. Kumar A. Kharwar R. N. (2007). Antagonistic potential of fluorescent pseudomonads and control of charcoal rot of chickpea caused by *Macrophomina phaseolina*. *J. Environ. Biol.* 28 15–20. 17717979

[B63] Lakhran L. Ahir R. R. Choudhary M. Choudhary S. (2018). Isolation, purification, identification and pathogenicity of *Macrophomina phaseolina* (Tassi) goid caused dry root rot of chickpea. *J. Pharmacogn. Phytochem.* 7 3314–3317.

[B64] Liu J. Blaylock L. A. Endre G. Cho J. Town C. D. VandenBosch K. A. (2003). Transcript profiling coupled with spatial expression analyses reveals genes involved in distinct developmental stages of an Arbuscular Mycorrhizal symbiosis. *Plant Cell* 15 2106–2123. 10.1105/tpc.014183 12953114 PMC181334

[B65] Liu J. Maldonado-Mendoza I. Lopez-Meyer M. Cheung F. Town C. D. Harrison M. J. (2007). Arbuscular mycorrhizal symbiosis is accompanied by local and systemic alterations in gene expression and an increase in disease resistance in the shoots. *Plant J.* 50 529–544. 10.1111/j.1365-313X.2007.03069.x 17419842

[B66] Lodha S. Sharma S. K. Aggarwal R. K. (1997). Solarization and natural heating of irrigated soil amended with cruciferous residues for improved control of *Macrophomina phaseolina*. *Plant Pathol.* 46 186–190. 10.1046/j.1365-3059.1997.d01-223.x

[B67] Lokesh R. Rakholiya K. B. Thesiya M. R. (2020). Evaluation of different fungicides against *Macrophomina phaseolina* (Tassi) goid. causing dry root rot of chickpea (*Cicer arietinum* L.) *in vitro*. *Artic. Int. J. Curr. Microbiol. Appl. Sci.* 9 1–11. 10.20546/ijcmas.2020.907

[B68] López-Ráez J. A. Verhage A. Fernández I. García J. M. Azcón-Aguilar C. Flors V. (2010). Hormonal and transcriptional profiles highlight common and differential host responses to arbuscular mycorrhizal fungi and the regulation of the oxylipin pathway. *J. Exp. Bot.* 61 2589–2601. 10.1093/jxb/erq089 20378666 PMC2882257

[B69] Lorenzetti E. Stangarlin J. R. Kuhn O. J. Portz R. L. (2018). Induction of resistance to *macrophomina phaseolina* in soyben treated with rosemary extract | Indução de resistência à *macrophomina phaseolina* em soja tratada com extrato de alecrim. *Summa Phytopathol.* 44 45–50. 10.1590/0100-5405/176895

[B70] Lygin A. V. Zernova O. V. Hill C. B. Kholina N. A. Widholm J. M. Hartman G. L. (2013). Glyceollin is an important component of soybean plant defense against Phytophthora sojae and *Macrophomina phaseolina*. *Phytopathology* 103 984–994. 10.1094/phyto-12-12-0328-r 23617338

[B71] Machado A. R. Pinho D. B. Soares D. J. Gomes A. A. M. Pereira O. L. (2019). Bayesian analyses of five gene regions reveal a new phylogenetic species of *Macrophomina* associated with charcoal rot on oilseed crops in Brazil. *Eur. J. Plant Pathol.* 153 89–100. 10.1007/s10658-018-1545-1

[B72] Mah K. M. Uppalapati S. R. Tang Y. Allen S. Shuai B. (2012). Gene expression profiling of *Macrophomina phaseolina* infected *Medicago truncatula* roots reveals a role for auxin in plant tolerance against the charcoal rot pathogen. *Physiol. Mol. Plant Pathol.* 79 21–30. 10.1016/j.pmpp.2012.03.004

[B73] Mahdizadeh V. Safaie N. Goltapeh E. (2012). Genetic diversity of sesame isolates of *Macrophomina phaseolina* using RAPD and ISSR markers. *Trakia J. Sci.* 10 65–74.

[B74] Mahdizadeh V. Safaie N. Goltapeh E. M. (2011). Diversity of *Macrophomina phaseolina* based on morphological and genotypic characteristics in Iran. *Plant Pathol. J.* 27 128–137. 10.5423/PPJ.2011.27.2.128

[B75] Mahmoud A. F. Abou-Elwafa S. F. Shehzad T. (2018). Identification of charcoal rot resistance QTLs in sorghum using association and in silico analyses. *J. Appl. Genet.* 59 243–251. 10.1007/s13353-018-0446-5 29876718

[B76] Marquez N. Giachero M. L. Gallou A. Debat H. J. Cranenbrouck S. Di Rienzo J. A. (2018). Transcriptional Changes in Mycorrhizal and Nonmycorrhizal soybean plants upon infection with the fungal pathogen *Macrophomina phaseolina*. *Mol. Plant. Microbe Interact.* 31 842–855. 10.1094/MPMI-11-17-0282-R 29498566

[B77] Marquez N. Giachero M. L. Gallou A. Debat H. J. Declerck S. Ducasse D. A. (2019). Transcriptome analysis of mycorrhizal and nonmycorrhizal soybean plantlets upon infection with *Fusarium virguliforme*, one causal agent of sudden death syndrome. *Plant Pathol.* 68 470–480. 10.1111/ppa.12964

[B78] Martinez-Medina A. Pozo M. J. Cammue B. P. A. Vos C. M. F. (2016). “Belowground defence strategies in plants: the plant–*Trichoderma* dialogue,” in *Belowground Defence Strategies in Plants*, eds Vos C. M. F. Kazan K. (Cham: Springer International Publishing), 301–327. 10.1007/978-3-319-42319-7_13

[B79] Mayék-Pérez N. López-Castañeda C. González-Chavira M. Garcia-Espinosa R. Acosta-Gallegos J. De La Vega O. M. (2001). Variability of Mexican isolates of *Macrophomina phaseolina* based on pathogenesis and AFLP genotype. *Physiol. Mol. Plant Pathol.* 59 257–264. 10.1006/pmpp.2001.0361

[B80] Mbaye N. Mame P. S. Ndiaga C. Ibrahima N. (2015). Is the recently described *Macrophomina pseudophaseolina* pathogenically different from *Macrophomina phaseolina*? *African J. Microbiol. Res.* 9 2232–2238. 10.5897/AJMR2015.7742

[B81] Mcloughlin A. G. Walker P. L. Wytinck N. Sullivan D. S. Whyard S. Belmonte M. F. (2018). Developing new RNA interference technologies to control fungal pathogens. *Can. J. Plant Pathol.* 40 325–335. 10.1080/07060661.2018.1495268

[B82] Mengistu A. Ray J. D. Smith J. R. Arelli P. R. Bellaloui N. Chen P. (2018). Effect of charcoal rot on selected putative drought tolerant soybean genotypes and yield. *Crop Prot.* 105 90–101. 10.1016/j.cropro.2017.11.012

[B83] Mmbaga M. T. Mackasmiel L. M. Mrema F. A. (2018). Evaluation of biological agents for control of *macrophomina* root rot and powdery mildew in flowering dogwood (*Cornus Florida* L.). *HortScience* 53 1461–1466. 10.21273/HORTSCI13071-18

[B84] Muchero W. Ehlers J. D. Close T. J. Roberts P. A. (2011). Genic SNP markers and legume synteny reveal candidate genes underlying QTL for *Macrophomina phaseolina* resistance and maturity in cowpea [*Vigna unguiculata* (L) Walp.]. *BMC Genomics* 12:8. 10.1186/1471-2164-12-8 21208448 PMC3025960

[B85] Nafady N. A. Hashem M. Hassan E. A. Ahmed H. A. M. Alamri S. A. (2019). The combined effect of arbuscular mycorrhizae and plant-growth-promoting yeast improves sunflower defense against *Macrophomina phaseolina* diseases. *Biol. Control* 138:104049. 10.1016/j.biocontrol.2019.104049

[B86] Oyewole B. O. Olawuyi O. J. Odebode A. C. Abiala M. A. (2017). Influence of *Arbuscular mycorrhiza* fungi (AMF) on drought tolerance and charcoal rot disease of cowpea. *Biotechnol. Rep.* 14 8–15. 10.1016/j.btre.2017.02.004 28459003 PMC5397100

[B87] Pandey A. K. Burlakoti R. R. Rathore A. Nair R. M. (2020). Morphological and molecular characterization of *Macrophomina phaseolina* isolated from three legume crops and evaluation of mungbean genotypes for resistance to dry root rot. *Crop Prot.* 127:104962. 10.1016/j.cropro.2019.104962

[B88] Paré P. W. Farag M. A. Krishnamachari V. Zhang H. Ryu C.-M. Kloepper J. W. (2005). Elicitors and priming agents initiate plant defense responses. *Photosynth. Res.* 85 149–159. 10.1007/s11120-005-1001-x 16075316

[B89] Parmar H. V. Kapadiya H. J. Bhaliya C. M. (2017). Efficacy of different fungicides against *Macrophomina phaseolina* (Tassi) Goid causing castor root rot. *Int. J. Chem. Stud.* 5 1807–1809.

[B90] Pawlowski M. L. Bowen C. R. Hill C. B. Hartman G. L. (2016). Responses of soybean genotypes to pathogen infection after the application of elicitors. *Crop Prot.* 87 78–84. 10.1016/j.cropro.2016.04.022

[B91] Pearson C. Leslie J. Schwenk F. (1987). Host preference correlated with chlorate resistance in *Macrophomina phaseolina*. *Plant Dis.* 71 828–831.

[B92] Perez-Brandán C. Arzeno J. L. Huidobro J. Grümberg B. Conforto C. Hilton S. (2012). Long-term effect of tillage systems on soil microbiological, chemical and physical parameters and the incidence of charcoal rot by *Macrophomina phaseolina* (Tassi) Goid in soybean. *Crop Prot.* 40 73–82. 10.1016/j.cropro.2012.04.018

[B93] Pozo M. J. Azcón-Aguilar C. (2007). Unraveling mycorrhiza-induced resistance. *Curr. Opin. Plant Biol.* 10 393–398. 10.1016/j.pbi.2007.05.004 17658291

[B94] Pozo M. J. Jung S. C. López-ráez J. A. (2010). “Impact of Arbuscular Mycorrhizal symbiosis on plant response to biotic stress: the role of plant defence mechanisms,” in *Arbuscular Mycorrhizas: Physiology and Function*, eds Koltai H. Kapulnik Y. (Cham: Springer), 193–207. 10.1007/978-90-481-9489-6

[B95] Purushotham N. Jones A. Poudel B. Nasim J. Adorada D. Sparks A. (2020). Draft genome resource for *Macrophomina phaseolina* associated with charcoal rot in sorghum. *Mol. Plant-Microbe Interact.* 33 724–726. 10.1094/MPMI-12-19-0356-A 32096690

[B96] Quazi M. M. H. Islam S. Emdadul M. E. Haque S. Alam M. Maqsudul A. (2019). Whole-genome optical mapping: improving assembly of *Macrophomina phaseolina* MS6 through spanning of twelve blunt end chromosomes by obviating all errors and misassembles. *African J. Biotechnol.* 18 1031–1043. 10.5897/ajb2019.16978

[B97] Rajpurohit T. S. (2002). Influence of intercropping and mixed cropping with pearl millet, green gram and mothbean on the incidence of stem and root rot (*Macrophomina phaseolina*) of sesame. *Sesame Safflower Newsl.* 17, 40–41.

[B98] Ramezani M. Shier W. T. Abbas H. K. Tonos J. L. Baird R. E. Sciumbato G. L. (2007). Soybean charcoal rot disease fungus *Macrophomina phaseolina* in Mississippi produces the Phytotoxin (-)-Botryodiplodin but no detectable Phaseolinone. *J. Nat. Prod.* 70 128–129. 10.1021/np060480t 17253865

[B99] Ramos A. M. Gally M. Szapiro G. Itzcovich T. Carabajal M. Levin L. (2016). In vitro growth and cell wall degrading enzyme production by Argentinean isolates of *Macrophomina phaseolina*, the causative agent of charcoal rot in corn. *Rev. Argent. Microbiol.* 48 267–273. 10.1016/j.ram.2016.06.002 27825736

[B100] Reznikov S. Chiesa M. A. Pardo E. M. De Lisi V. Bogado N. González V. (2019). Soybean-*Macrophomina phaseolina*-Specific interactions and identification of a novel source of resistance. *Phytopathology* 109 63–73. 10.1094/PHYTO-08-17-0287-R 30009663

[B101] Reznikov S. Vellicce G. R. González V. de Lisi V. Castagnaro A. P. Ploper L. D. (2016). Evaluation of chemical and biological seed treatments to control charcoal rot of soybean. *J. Gen. Plant Pathol.* 82 273–280. 10.1007/s10327-016-0669-4

[B102] Reznikov S. Vellicce G. R. Mengistu A. Arias R. S. Gonzalez V. De Lisi V. (2018). Disease incidence of charcoal rot (*Macrophomina phaseolina*) on soybean in north-western Argentina and genetic characteristics of the pathogen. *Can. J. Plant Pathol.* 40 423–433. 10.1080/07060661.2018.1484390

[B103] Rivero J. Gamir J. Aroca R. Pozo M. J. Flors V. (2015). Metabolic transition in mycorrhizal tomato roots. *Front. Microbiol.* 6:598. 10.3389/fmicb.2015.00598 26157423 PMC4477175

[B104] Saima S. Wu G. (2019). Effect of *Macrophomina phaseolina* on growth and expression of defense related genes in *Arabidopsis thaliana*. *J. Natl. Sci. Found. Sri Lanka* 47:113. 10.4038/jnsfsr.v47i1.8934

[B105] Salvatore M. M. Félix C. Lima F. Ferreira V. Naviglio D. Salvatore F. (2020). Secondary metabolites produced by *Macrophomina phaseolina* isolated from eucalyptus globulus. *Agriculture* 10:72. 10.3390/agriculture10030072

[B106] Sanjeevkumar K. Kumar K. S. Balabaskar P. Sivakumar T. Kannan R. Saravanan K. R. (2020). Efficacy of seed plus soil application of Bacillus cereus on the root rot (*Macrophomina phaseolina* (tassi.) Goid.) incidence and plant growth of groundnut. *Plant Arch.* 20 1217–1221.

[B107] Santos K. M. Lima G. S. Barros A. P. O. Machado A. R. Souza-Motta C. M. Correia K. C. (2020). Novel specific primers for rapid identification of *Macrophomina* species. *Eur. J. Plant Pathol.* 156 1213–1218. 10.1007/s10658-020-01952-8

[B108] Sarkar T. S. Biswas P. Ghosh S. K. Ghosh S. (2014). Nitric oxide production by necrotrophic pathogen *Macrophomina phaseolina* and the host plant in charcoal rot disease of jute: complexity of the interplay between necrotroph-host plant interactions. *PLoS One* 9:e0107348. 10.1371/journal.pone.0107348 25208092 PMC4160249

[B109] Sarr M. P. Ndiaye M. Groenewald J. Z. Crous P. W. (2014). Genetic diversity in *Macrophomina phaseolina*, the causal agent of charcoal rot. *Phytopathol. Mediterr.* 53 250–268. 10.14601/Phytopathol_Mediterr-13736

[B110] Schroeder M. M. Lai Y. Shirai M. Alsalek N. Tsuchiya T. Roberts P. (2019). A novel Arabidopsis pathosystem reveals cooperation of multiple hormonal response-pathways in host resistance against the global crop destroyer *Macrophomina phaseolina*. *Sci. Rep.* 9:20083. 10.1038/s41598-019-56401-2 31882671 PMC6934584

[B111] Sharma I. Kumari N. Sharma V. (2014). Defense gene expression in Sorghum bicolor against *Macrophomina phaseolina* in leaves and roots of susceptible and resistant cultivars. *J. Plant Interact.* 9 315–323. 10.1080/17429145.2013.832425

[B112] Sharma R. C. Bhowmik T. P. (1986). Estimation of yield losses in groundnut due to *Macrophomina phaseolina* (Tassi) Goid. *Indian J. Plant Pathol.* 4 108–112.

[B113] Short G. E. Wyllie T. D. Ammon V. D. (1978). Quantitative enumeration of *Macrophomina phaseolina* in soybean tissues. *Phytopathology* 68 736–741.

[B114] Simonetti E. Viso N. P. Montecchia M. Zilli C. Balestrasse K. Carmona M. (2015). Evaluation of native bacteria and manganese phosphite for alternative control of charcoal root rot of soybean. *Microbiol. Res.* 180 40–48. 10.1016/j.micres.2015.07.004 26505310

[B115] Sinclair J. B. Backman P. A. (1989). *Compendium of Soybean Diseases*, 3rd Edn. St.Paul, MN: APS Press.

[B116] Smith G. S. Carvil O. N. (1997). Field screening of commercial and experimental soybean cultivars for their reaction to *Macrophomina phaseolina*. *Plant Dis.* 81 363–368. 30861816 10.1094/PDIS.1997.81.4.363

[B117] Spagnoletti F. Carmona M. Gómez N. E. T. Chiocchio V. Lavado R. S. (2017). Arbuscular mycorrhiza reduces the negative effects of *Macrophomina phaseolina* on soybean plants in arsenic-contaminated soils. *Appl. Soil Ecol.* 121 41–47. 10.1016/j.apsoil.2017.09.019

[B118] Spagnoletti F. N. Cornero M. Chiocchio V. Lavado R. S. Roberts I. N. (2020). Arbuscular mycorrhiza protects soybean plants against *Macrophomina phaseolina* even under nitrogen fertilization. *Eur. J. Plant Pathol.* 156 839–849. 10.1007/s10658-020-01934-w

[B119] Spagnoletti F. N. Leiva M. Chiocchio V. Lavado R. S. (2018). Phosphorus fertilization reduces the severity of charcoal rot (*Macrophomina phaseolina*) and the arbuscular mycorrhizal protection in soybean. *J. Plant Nutr. Soil Sci.* 181 855–860. 10.1002/jpln.201700569

[B120] Sridharan A. Thankappan S. Karthikeyan G. Uthandi S. (2020). Comprehensive profiling of the VOCs of *Trichoderma longibrachiatum* EF5 while interacting with Sclerotium rolfsii and *Macrophomina phaseolina*. *Microbiol. Res.* 236:126436. 10.1016/j.micres.2020.126436 32179388

[B121] Srinivasa Reddy P. Fakrudin B. Rajkumar B. K. Punnuri S. M. Arun S. S. Kuruvinashetti M. S. (2007). Molecular mapping of genomic regions harboring QTLs for stalk rot resistance in sorghum. *Euphytica* 159 191–198. 10.1007/s10681-007-9472-9

[B122] Swamy C. Naik M. K. Amaresh Y. S. Jayalakshmi S. K. (2018). Evaluation of Fungicides and Bio-Agents under *in vitro* Condition against *Macrophomina phaseolina* causing stem canker of Pigeonpea. *Int. J. Curr. Microbiol. Appl. Sci.* 7 811–819. 10.20546/ijcmas.2018.701.099

[B123] Tančić Živanov S. Dedić B. Dimitrijević A. Dušanić N. Jocić S. Miklič V. (2019). Analysis of genetic diversity among *Macrophomina phaseolina* (Tassi) Goid. isolates from Euro-Asian countries. *J. Plant Dis. Prot.* 126 565–573. 10.1007/s41348-019-00260-6

[B124] Tok F. M. (2019). Relationship between morphologic, phenotypic and pathogenic characteristics in *Macrophomina phaselina* Isolates from cucumber plants. *Int. J. Innov. Approaches Agric. Res.* 3 651–660. 10.29329/ijiaar.2019.217.11

[B125] Tomar R. S. Parakhia M. V. Rathod V. M. Thakkar J. Padhiyar S. M. Thummar V. D. (2017). Molecular mapping and identification of QTLs responsible for charcoal rot resistance in Castor (*Ricinus communis* L.). *Ind. Crops Prod.* 95 184–190. 10.1016/j.indcrop.2016.10.026

[B126] Tonin R. F. B. Avozani A. Durante Danelli A. L. Reis E. M. Zoldan S. M. Garcés-Fiallos F. R. (2013). *In vitro* mycelial sensitivity of *macrophomina phaseolina* to fungicides | Sensibilidade micelial in vitro de *macrophomina phaseolina* a fungicidas. *Pesqui. Agropecu. Trop.* 43 460–466. 10.1590/S1983-40632013000400014

[B127] Torres M. J. Brandan C. P. Petroselli G. Erra-Balsells R. Audisio M. C. (2016). Antagonistic effects of Bacillus subtilis subsp. subtilis and B. amyloliquefaciens against *Macrophomina phaseolina*: SEM study of fungal changes and UV-MALDI-TOF MS analysis of their bioactive compounds. *Microbiol. Res.* 182 31–39. 10.1016/j.micres.2015.09.005 26686611

[B128] Ugulino A. L. N. Mendonça Júnior A. F. Rodrigues A. P. M. Santos A. França K. Cardoso T. (2018). Inhibition effect of vegetable oils on the mycelial growth of *Macrophomina phaseolina* (Tassi.). *Goid. J. Agric. Sci.* 10:49. 10.5539/jas.v10n6p49

[B129] Ushiki J. Hayakawa Y. Tadano T. (1996). Medicinal plants for suppressing soil-borne plant diseases: I. Screening for medicinal plants with antimicrobial activity in roots. *Soil Sci. Plant Nutr.* 42 423–426. 10.1080/00380768.1996.10415116

[B130] Vasebi Y. Safaie N. Alizadeh A. (2013). Biological control of soybean charcoal root rot disease using bacterial and fungal antagonists *in vitro* and greenhouse condition. *J. Crop Prot.* 2, 139–150.

[B131] Vimal S. R. Singh J. S. Arora N. K. Singh S. (2017). Soil-Plant-microbe interactions in stressed agriculture management: a review. *Pedosphere* 27 177–192. 10.1016/S1002-0160(17)60309-6

[B132] Wang J. Xiao Y. Liu X. Ni Y. Zhao H. Zhao X. (2019a). Complete genome sequence of a novel victorivirus isolated from the sesame charcoal rot fungus *Macrophomina phaseolina*. *Arch. Virol.* 165 509–514. 10.1007/s00705-019-04497-2 31845152

[B133] Wang J. Xiao Y. Zhao H. Ni Y. Liu X. Zhao X. (2019b). A novel double-stranded RNA mycovirus that infects *Macrophomina phaseolina*. *Arch. Virol.* 164 2411–2416. 10.1007/s00705-019-04334-6 31254049

[B134] Wrather A. Shannon G. Balardin R. Carregal L. Escobar R. Gupta G. K. (2010). Effect of diseases on soybean yield in the top eight producing countries in 2006. *Plant Heal. Prog.* 10 2008–2013. 10.1094/PHP-2010-0125-01-RS

[B135] Wyllie T. D. (1988). “Charcoal rot of soybeans-current status,” in *Soybean Diseases of the North Central Region*, eds Wyllie T. D. DH S. (St. Paul, MN: APS Press), 106–113.

[B136] Xie J. Jiang D. (2014). New insights into mycoviruses and exploration for the biological control of crop fungal diseases. *Annu. Rev. Phytopathol.* 52 45–68. 10.1146/annurev-phyto-102313-050222 25001452

[B137] Zaman N. R. Kumar B. Nasrin Z. Islam M. R. Maiti T. K. Khan H. (2020). Proteome analyses reveal *Macrophomina phaseolina* ’s survival tools when challenged by *Burkholderia contaminans* NZ. *ACS Omega* 5 1352–1362. 10.1021/acsomega.9b01870 32010805 PMC6990438

[B138] Zaynab M. Fatima M. Abbas S. Sharif Y. Umair M. Zafar M. H. (2018). Role of secondary metabolites in plant defense against pathogens. *Microb. Pathog.* 124 198–202. 10.1016/j.micpath.2018.08.034 30145251

[B139] Zhang Q. Gao X. Ren Y. Ding X. Qiu J. Li N. (2018). Improvement of *Verticillium* wilt resistance by applying arbuscular mycorrhizal fungi to a cotton variety with high symbiotic efficiency under field conditions. *Int. J. Mol. Sci.* 19:241. 10.3390/ijms19010241 29342876 PMC5796189

